# In the Heart of the Amazon: Noncommunicable Diseases and Apolipoprotein E4 Genotype in the Riverine Population

**DOI:** 10.3390/ijerph15091957

**Published:** 2018-08-13

**Authors:** Gabriela P. F. Arrifano, Jacqueline I. Alvarez-Leite, José Rogério Souza-Monteiro, Marcus Augusto-Oliveira, Ricardo Paraense, Barbarella M. Macchi, André Pinto, Reinaldo B. Oriá, José Luiz Martins do Nascimento, Maria Elena Crespo-Lopez

**Affiliations:** 1 Laboratório de Farmacologia Molecular, Instituto de Ciências Biológicas, Universidade Federal do Pará, Belém 66075-110, Brazil; gabrielaarrifano@uol.com.br (G.P.F.A.); rogerio.souza.monteiro@gmail.com (J.R.S.-M.); pos_ricardo@hotmail.com (R.P.); 2 Departamento de Bioquímica e Imunologia, Universidade Federal de Minas Gerais, Belo Horizonte 31270-90, Brazil; jalvarezleite@gmail.com; 3 Laboratório de Investigações em Neurodegeneração e Infecção (Hospital Universitário João de Barros Barreto), Universidade Federal do Pará, Belém 66063-023, Brazil; marcusoliveira@globo.com; 4 Laboratório de Neuroquímica e Biologia Celular, Instituto de Ciências Biológicas, Universidade Federal do Pará, Belém 66075-110, Brazil; barbarella@ufpa.br (B.M.M.); jlmn@ufpa.br (J.L.M.d.N.); 5 Laboratório de Análises Clínicas, Instituto de Ciências Biológicas, Universidade Federal do Pará, Belém 66075-110, Brazil; andremontep1@hotmail.com; 6 Laboratório da Biologia da Cicatrização, Ontogenia e Nutrição de Tecidos, Departamento de Morfologia e Instituto de Biomedicina, Escola de Medicina, Universidade Federal do Ceará, Fortaleza 60430-160, Brazil; oria@ufc.br

**Keywords:** Amazon, hypertension, diabetes, noncommunicable diseases, apolipoprotein, apoE, genotyping, riverine, Tapajos, Tucurui

## Abstract

The Amazon River basin is the largest tropical forest in the world. Most of the Amazon belongs to Brazil, a developing country that currently faces huge challenges related to the consolidation of its universal healthcare system. Noncommunicable diseases (NCDs) are the leading cause of death in Brazil, accounting for 74% of all deaths, and NCDs are probably underestimated in Amazonian population because of their geographical isolation and the precariousness of riverine communities. Important risk factors, such as genetic susceptibility, remain undetermined in the riverine population. This study performed fasting blood sugar (FBS) and blood pressure measurements and investigated the presence of the ε4 allele of apolipoprotein E (APOE4) to determine the prevalence of diabetes, hypertension and the genetic risk of NCDs. FBS and APOE4 were measured in blood samples from 763 participants using spectrometry and real-time PCR; 67.5% showed altered measurements, and 57.9% had never been diagnosed or treated. Altered FBS was found in 28.3% of the participants, hypertension in 57.6% and APOE4 in 32.0%. The health profile of the riverine population appears to differ from that of urban population in the Amazon. Additional risk factors for NCDs, such as environmental contamination and nutritional transition, may contribute more than increased genetic susceptibility to the prevalence of altered FBS and hypertension. Our results will help guide the development of preventive strategies and governmental actions for more effective management of NCDs in the Amazon area.

## 1. Introduction

The Amazon River basin is the largest tropical forest in the world and is home to more than 17 million people [1]. Most of the Amazon belongs to Brazil, a developing country with an emerging economy that faces huge challenges related to the consolidation of its universal healthcare system [2]. The disparities between the richest 10% and the poorest 10% of the Brazilian population have grown in recent decades [2], and the Northern region has the lowest Human Development Index [3].

In the Amazon, a large part of the population lives far from major cities in small riverine communities or in widespread family-based houses. The Amazonian riverine population is mixed-ethnicity (Indigenous/Amerindian/Portuguese/African) [4], and the Amazon river is a central element in the lifestyle of the population. Fish are the main source of protein in the diet and are usually consumed in more than seven meals per week. Water from the river or from hand-dug wells is used for cooking, and there is a risk of water contamination by garbage that is burned, buried, or dumped directly into the river. Conditions are not always sanitary, as there is no electricity or sewage system, and the population has little or no access to medical facilities. Most of the population goes to school for eight years or less. Although a wide variety of fruits are available and are widely consumed [5], dietary factors such as the nutrition transition [5,6] may be contributing to an increase in the prevalence of noncommunicable diseases (NCDs) in these communities. The area’s geographical isolation and poor access to healthcare make it difficult to routinely collect epidemiological data about health problems such as NCDs in these communities.

The most prevalent NCDs are cardiovascular diseases (CVDs), chronic respiratory diseases, diabetes mellitus (DM), and cancer. NCDs cause approximately 40 million deaths worldwide annually and are one of the major health and development challenges of the 21st century [7]. About 80% of all premature deaths (30–69 years) in the world due to NCDs occur in low- and middle-income countries [7], most of them related to cardiovascular conditions.

Determining the presence of the ε4 allele of apolipoprotein E (*APOE4* for the gene; ApoE4 for the protein) is important for evaluating the risk of NCDs [8,9,10]. Apolipoprotein E (ApoE) is a 299-amino acid, 34-kD glycoprotein that plays an essential role in the regulation of lipid metabolism and in cholesterol transport [11,12]. Humans have three major isoforms of this apolipoprotein due to single nucleotide polymorphisms at positions 112 and 158: ApoE2 (Cys112/Cys158), ApoE3 (Cys112/Arg158), and ApoE4 (Arg112/Arg158). These minor differences in the primary structures of the ApoE isoforms have major effects on both its secondary structure and its function. *APOE4* is a recognized genetic risk factor for CVDs and for age-related NCDs, especially neurodegenerative NCDs such as dementia. Recent works in humans found associations between ApoE4 and diverse NCDs, such as DM, dyslipidemias, and thrombosis [8,9,13,14]. Animal studies have established some of the molecular mechanisms underlying these associations. For example, ApoE4 may confer a proatherogenic profile by impairing VLDL clearance because ApoE4 has a greater lipid and VLDL-binding ability than ApoE3 [15]. ApoE4 may also impair insulin signaling in the brain, probably because its direct binding to the insulin receptor may trap it in endosomes preventing it from interacting with insulin [16].

In Brazil, NCDs are the leading cause of death, accounting for 74% of all deaths in the country [17,18]. The prevalence of chronic diseases, including hypertension and DM, is worryingly high—for example, Brazil is currently ranked as the fourth country in the world in terms of the number of patients with DM [19,20]. The most recent data indicates that the prevalence of medical diagnosis of hypertension and DM have increased 3.5% and 2.8%, respectively, in the last decade [18]. However, in some regions, such as the Amazon River basin, the prevalence of NCDs is probably underestimated due to geographical isolation and the precariousness of healthcare for people living in the riverine communities. Thus, the Amazon area represents a huge healthcare challenge. In addition, the prevalence of important risk factors, such as those associated with possible genetic susceptibility, is not totally known in these populations.

Given this context, we investigated fasting blood sugar (FBS) levels, blood pressure, and the presence of ApoE4 in Amazonian riverine population in order to determine the prevalence of DM and hypertension and to evaluate the risk of NCDs.

## 2. Materials and Methods

### 2.1. Ethics

This project followed the guidelines of the Declaration of Helsinki and was approved by the National Council for Ethics in Research (CONEP, Brazil; CAAE no. 43927115.4.0000.0018). The participants were informed about the aim of the study prior to their inclusion and provided written consent for participation in the study.

### 2.2. Study Population and Inclusion/Exclusion Criteria

**Figure 1:**
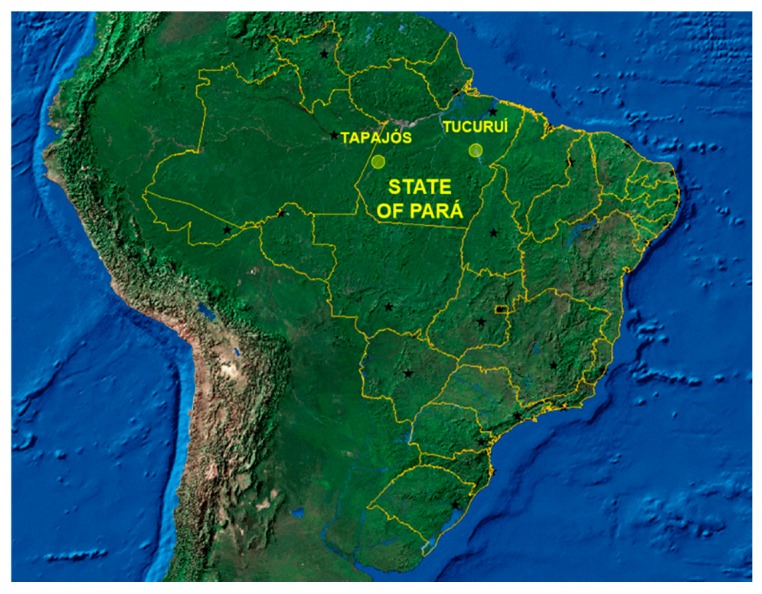
Map of the States of Brazil (yellow lines) and their capitals (black stars) obtained from the Instituto Brasileiro de Geografía e Estatística (IBGE, Brazil). The State of Pará and the approximate locations of the two regions (Tapajós and Tucuruí) are identified.

The Amazonian population in this study was from the State of Pará, Brazilian Amazon (Figure 1). Two regions were included that represented the two most common social structures found in Amazonian riverine populations: the Tapajós River basin (−4.287121, −55.984106), which comprises small communities that live alongside the river; and Tucuruí Lake (−3.800897, −49.811848) in which the riverine inhabitants live on islands, with a few families on each island.

This project was publicized via radio, meetings, and direct communications with healthcare agents. Samples were collected from volunteer participants at community meeting places such as schools. The inclusion criteria included adults ≥18 years old living in Amazonian riverine communities for at least two years. Participants without venous access, who registered twice, or who refused to donate blood were excluded. The first statistical analysis was performed using these inclusion/exclusion criteria (Analysis 1—NCDs in the total population).

Additionally, to better study the ‘presumably healthy’ population, a second statistical analysis (Analysis 2—NCDs in the ‘presumably healthy’ population) was performed to evaluate a subgroup of participants that was created *a posteriori* by using additional exclusion criteria: >65 years old; a history or previous diagnosis of an NCD; drug dependency, including tobacco and alcohol; and chronic treatment with drugs.

The *APOE4* genotype risk factor was analyzed statistically for the total population and for the subgroup of ‘presumably healthy’ participants (Analysis 3—Genetic risk factor (APOE4) for NCDs).

### 2.3. Sample and Data Collection

Data collection was performed from 2015 to 2017. A questionnaire was administered to each participant by personnel that were trained to register data related to health conditions, including the presence of pre-existing chronic diseases (such as CVDs, hypertension or DM), drug dependency (including tobacco and alcohol), and chronic treatments with drugs.

To quantify supine systolic and diastolic blood pressure (SBP and DBP, respectively), at least two measures were performed in the left arm separated by a 10-min rest interval, and then the mean was calculated. An approximately 3 mL of fasting blood sample was collected by venipuncture: 2 mL were collected into a vacutainer EDTA tube and immediately frozen and stored until the isolation of genomic DNA, and 1 mL was collected into a BD Vacutainer SST II Advance^®^ tube, which was immediately processed for FBS determination. Serum was separated by centrifugation of the 1-mL sample, and FBS was assayed with a commercial kit (Labtest, Belém, Brazil).

### 2.4. Detection of the Apolipoprotein E4 Genotype (APOE4)

Genomic DNA was isolated from the frozen blood samples using the Purelink Genomic DNA Mini Kit (ThermoFisher, São Paulo, Brazil), according to the manufacturer’s instructions. The presence of *APOE4* was detected by real time-PCR using TaqMan^®^ assays (ThermoFisher, São Paulo, Brazil) using StepOnePlus^®^ equipment (ThermoFisher, São Paulo, Brazil). Two *APOE* single-nucleotide polymorphisms (SNPs), rs429358 and rs7412, were analyzed as described elsewhere [4]. All materials, including primers and probes, were obtained from ThermoFisher (São Paulo, Brazil). The reactions were carried out in 96-well microtiter plates. The assay volume was 10 µL: 5 µL of 2X TaqMan Universal PCR Master Mix (ThermoFisher, São Paulo, Brazil), 25 nmol/L of each probe (FAM or VIC-labeled), and 0.5 µL of DNA (30–100 ng). Reactions were performed in duplicate with 40 cycles of denaturation at 92 °C for 15 s and hybridization and extension at 60 °C for 1 min.

### 2.5. Statistical Analysis

Statistical analysis was performed using GraphPad Prism 6 software (available at: www.graphpad.com). The Gaussian distribution of the data was analyzed using the D’Agostino–Pearson omnibus normality test. Nonparametric data are reported as medians and interquartile ranges, and the Mann-Whitney *U* test was used to detect differences between two groups. Frequencies were analyzed with Fisher’s exact test, the Chi-square test, and binomial test. Hardy-Weinberg equilibrium was tested to verify polymorphisms in *APOE* gene. For all analyses, *p* < 0.05 was considered statistically significant.

## 3. Results

### 3.1. Analysis 1—NCDs in the Total Population

A total of 857 people registered for our study, 94 of whom were excluded because they did not have venous access, participated twice, or refused to donate blood. Complete data were available for 763 adult participants (487 women and 276 men). The participants’ demographic and clinical characteristics are reported in Table 1.

The median BMI was 26.0 kg/m^2^, ranging from 16.6 to 48.6 kg/m^2^ (Table 1). Women had higher BMI than men. The median FBS was 90 mg/dL, ranging from 32 to 359 mg/dL; the median SBP was 125 mmHg, ranging from 70 to 239 mmHg; and the median DBP was 79 mmHg, ranging from 44 to 131 mmHg (Table 1). Men had higher SBP and DBP values than women, but there were no significant differences in FBS levels according to sex (Table 1).

The American Diabetes Association (2016) guidelines [21] were used to determine which participants had altered FBS: 100–125 mg/dL was considered impaired glucose tolerance and ≥126 mg/dL was considered DM. Because a DM diagnosis requires at least two measures of FBS ≥126 mg/dL, patients with a single measure of FBS ≥ 126 mg/dL were categorized as “DM-suspected”. The American College of Cardiology and the American Heart Association (ACC/AHA) guidelines [22] were used to identify individuals with altered blood pressure, so systemic arterial hypertension (SAH) was defined as SBP ≥ 130 mmHg and/or DPB ≥ 80 mmHg.

**Figure 2:**
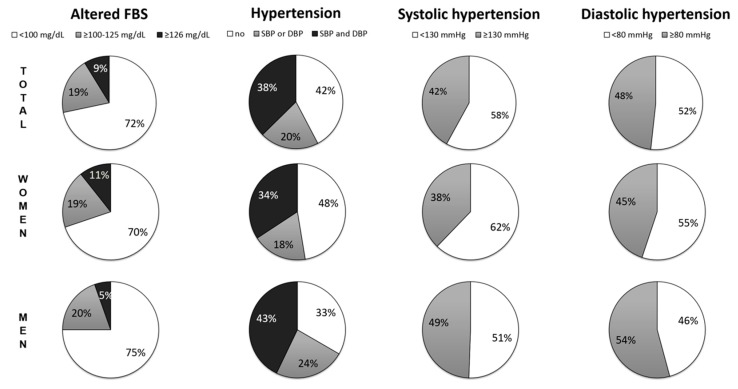
The prevalence of altered fasting blood sugar (FBS), hypertension, systolic hypertension or diastolic hypertension in the total population (first line), in women (second line), and in men (third line). According to the American Diabetes Association (2016) [21], impaired glucose tolerance was defined as FBS ≥100–125 mg/dL (gray) and diabetes mellitus (DM)-suspected was defined as ≥126 mg/dL (black). Systolic hypertension was defined as blood pressure ≥130 mmHg and diastolic hypertension as blood pressure ≥80 mmHg.

Surprisingly, 515 participants (67.5% (95% confidence interval, CI: 64.0–70.8)) showed altered FBS (impaired glucose tolerance or DM-suspected) and/or SAH. Indeed, 134 individuals (17.8%; CI: 15.1–20.7) had both SAH and altered FBS. The prevalence of DM-suspected was 10.7% (CI: 8.1–13.8) in women; this was significantly higher than in men (5.4%; CI: 3.1–8.8) (Fisher’s exact test, *p* < 0.05) (Figure 2). In both women and men, the prevalence of hypertension (systolic or diastolic) was significantly higher than that of altered FBS (Fisher’s exact test, *p* < 0.0001) (Figure 2). However, men more frequently had SAH (66.5%; CI: 60.6–72.1) than women (52.6%; CI: 48.0–57.2) (Fisher’s exact test, *p* < 0.001) (Figure 2).

### 3.2. Analysis 2—NCDs in the ‘Presumably Healthy’ Sub-Group

**Figure 3:**
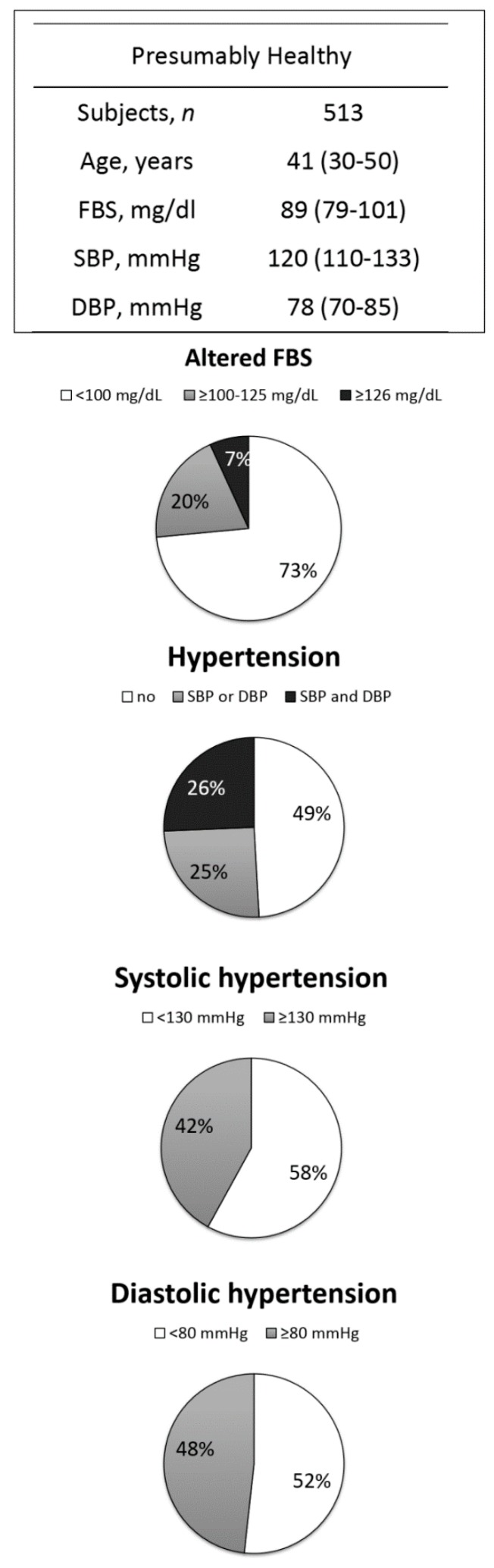
The prevalence of altered fasting blood sugar (FBS) levels, systolic hypertension, and diastolic hypertension in the presumably healthy subgroup. The presumably healthy subgroup was defined as participants who were 18–65 years old with no history or previous diagnosis of NCDs, drug dependency, or chronic treatment with drugs. According to the American Diabetes Association (2016) [21], impaired glucose tolerance was defined as FBS ≥100–125 mg/dL (gray) and diabetes mellitus (DM)-suspected was defined as ≥126 mg/dL (black). Systolic hypertension was defined as blood pressure ≥130 mmHg and diastolic hypertension as blood pressure ≥80 mmHg. Data are presented as medians and interquartile ranges (table) and as proportions of participants (graphs).

We used additional exclusion criteria to identify and analyze a ‘presumably healthy’ population. This was a subgroup of participants who were 18–65 years old with no history or previous diagnosis of NCDs, drug dependency, or chronic treatment with drugs. This subgroup (*n* = 513) was younger and showed median values of all parameters slightly lower than the total population (Figure 3). Surprisingly, a high proportion (58.1%; CI: 53.7–62.4; *n* = 298) of participants of this ‘presumably healthy’ subgroup had values above the reference limits despite the inclusion/exclusion criteria. Specifically, the prevalence of altered FBS was 26.5% (CI: 22.7–30.6) and the prevalence of SAH (systolic and/or diastolic) was 45.2% (CI: 40.8–49.6) (Figure 3).

### 3.3. Analysis 3—Genetic Risk Factor (APOE4) for NCDs

**Figure 4:**
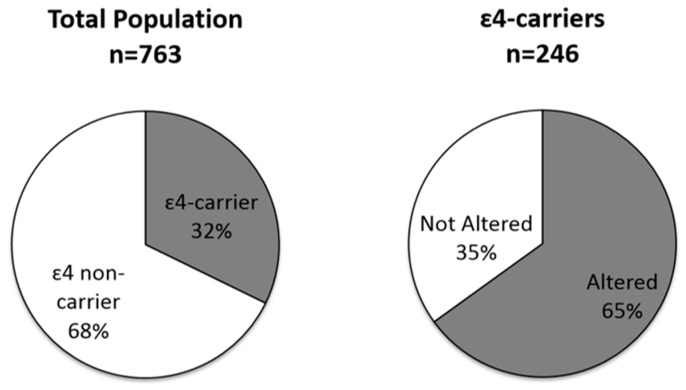
The distribution of *APOE4* in the total population. The percentage of ɛ4 carriers in the total population (**left**, gray) and the proportion of ɛ4 carriers showing altered fasting blood sugar (≥100 mg/dL) and/or systemic arterial hypertension (**right**, gray).

A substantial number of participants carried the ε4 allele of the *APOE* gene (Figure 4), and many already showed alterations. Data from the total population was in Hardy-Weinberg equilibrium (χ^2^ = 0.3625). The most frequent *APOE* genotypes were ε3/ε3 (59.5%) and ε3/ε4 (26.5%). The allelic frequencies were 0.05, 0.77, and 0.18 for the ε2, ε3 and ε4 alleles, respectively. The proportion of participants with high FBS (≥100 mg/dL) and/or SAH was similar for the ε4 carriers (65.0%; CI: 58.7–71.0) and the non-ε4 carriers (68.7%; CI: 64.5–72.6) (Fisher’s exact test, *p* > 0.05). Notably, 166 individuals (32.4%; CI: 28.3–36.6) in the ‘presumably healthy’ subgroup were ε4 carriers, while 100 individuals who were ε4 carriers (60.2%; CI: 52.4–67.7) in the ‘presumably healthy’ subgroup had altered FBS (≥100 mg/dL) and/or SAH.

## 4. Discussion

This study is the first to evaluate the prevalence of two NCDs and a genetic risk factor in Amazonian riverine population. It found a high prevalence of altered FBS (impaired glucose tolerance or DM-suspected) and/or SAH. More than half of the individuals with altered FBS and/or SAH did not have a previous diagnosis and were not being treated. In addition, a high proportion of the participants were ε4 carriers, and 65.0% of these individuals already had altered FBS and/or hypertension.

Age-adjusted NCD mortality has fallen by 1.8% per year since the implementation of policies to prevent NCDs in Brazil [23]. However, the NCDs like CVDs continue to have deleterious consequences. Recent data indicate that although the mortality rate of some CVDs, such as heart ischemia, seem to be steady in Brazil, this is due to a decrease in the number of deaths due to cardiovascular causes in the south and southeast regions that is balanced by significant increases in deaths in the northern–northeastern region [24]. Between 2000 and 2010, the number of deaths increased by 28% in the northern region, but decreased by 13% to 25% in the southern–southeastern regions. We hypothesize that this significant increase in the northern region is underestimated because the communities are typically isolated, leading to underreporting of NCDs. Two observations support our hypothesis. First, Amazonian cities report a low prevalence of SAH and diabetes—indeed, the reported prevalence of these conditions is among the lowest in all Brazilian capital cities [18]. Second, our data showed that the prevalence in riverine population was as high as the prevalence in São Paulo and Rio de Janeiro, the largest cities in the country.

This study included 763 participants (Table 1), it was similar in size or larger than published studies that have looked at the characteristics of the Amazonian riverine population [25,26,27,28,29,30,31,32,33,34,35].

In Brazil, the probability of dying between 30 and 70 years of age because of an NCD is 19% [17]. The median age of the population in our study was 47 years, with 575 individuals (75.4%; 72.1–78.4) between 30 and 70 years of age. Surprisingly, 71.3% (CI: 67.4–75.0) of these 575 individuals had FBS ≥100 mg/dL and/or hypertension. These data suggest that there is an urgent need for interventions in these populations. According to the WHO (2014) [17], four main NCD risk factors are modifiable: unhealthy diets, physical inactivity, tobacco use, and the harmful use of alcohol. Eliminating these risk factors could prevent 80% of all cases of heart disease, stroke, and type 2 diabetes and more than 40% of cases of cancer [17].

Diabetes diagnosis in Brazilian capitals increased from 5.5% to 7.4% in the last 10 years [18]. Interestingly, educational level has been associated with diabetes prevalence, with individuals with 0–8 years of formal education having a more than three-fold higher prevalence (13.5%) compared to those with >8 years of education (4.4%) [18]. Educational level might also be associated with a higher prevalence of diabetes in isolated Amazonian populations, such as those in riverine communities (Figure 2), who traditionally have a low educational level.

Impaired glucose tolerance is a major risk factor for type 2 diabetes [9], so the high number of participants in our study with impaired glucose tolerance (*n* = 149; 19%) is a serious concern (Figure 2). If these individuals do not change their habits and if they continue to be inadequately treated, they could develop type 2 diabetes in the near future. Strikingly, in addition to the 9% of individuals that were classified as DM-suspected, this means that as many as 28% of the riverine population could develop DM in the future. DM can result in irreversible visual impairment (i.e., blindness), in the amputation of extremities, and it can lead to premature retirement. Thus, the prevalence could exceed the current prevalence of DM in urban populations of the Amazon [18,33].

The difference in the number of women and men who participated in our study was expected, since this imbalance is common in epidemiological studies of Amazonian populations [35,36]. Women in these communities seem to be more careful with their healthcare than men, leading to this imbalance [37]. The role that male versus female sex plays in the prevalence of NCDs is still a matter of debate. Many studies support the notion that the prevalence of NCDs differs according to sex [38,39]. A survey of subjects in middle- and low-income countries reported that male sex is a risk factor for NCDs [40]. Interestingly, recent data from a large cohort of subjects in the Taiwanese military support this hypothesis, demonstrating that high blood pressure (among other components of metabolic syndrome) was more prevalent in men than in women [39]. Our data may support this for SAH, because men had significantly higher median SBP and DBP values and hypertension than women (Table 1 and Figure 2). However, additional studies are needed to confirm this hypothesis, since there may be other contributing factors. For example, the men in this study were older than the women (Table 1), and the risk of hypertension increases with increased age [41]. On the other hand, women had significantly higher BMI values than men (Table 1), and central obesity plays an important role in the development of NCDs [23].

Our results in women of riverine communities showed that women were more frequently affected by SAH than by altered FBS, based on the 2017 ACC/AHA guidelines [22] (Figure 2). However, the previous AHA and VIGITEL (2016) guidelines [18,42] defined SAH as SBP ≥ 140 mmHg and/or DPB ≥ 90 mmHg rather than 130 and 80, respectively. Using the latter guidelines, the prevalence of altered FBS in women, 30%, was higher than the prevalence of hypertension (systolic or diastolic), 22 to 20%, (Fisher’s exact test, *p* < 0.05). Notably, in Amazonian urban populations, SAH showed a higher prevalence in women [18,33]. Environmental factors, nutritional factors, and life style factors contribute to the differences in NCD profiles in urban and riverine populations of the Amazon, emphasizing the importance of conducting epidemiological studies.

The most recent data [18] show that diagnosis of SAH have increased by 3.5% in the last 10 years in Brazilian capitals, with higher prevalence in women and in individuals with a low educational level. Curiously, the northern region currently shows the lowest SAH prevalence in the country [18]. Hypertension is a preventable cause of cardiovascular morbidity and mortality, and blood pressure is a good indicator of risk for CVDs. This parameter is relatively easy to measure, even in remote field research conditions [26], but few studies have investigated blood pressure in Amazonian riverine populations.

Interestingly, the Tapajós River Basin is one of the few locations in the Amazon in which blood pressure has been evaluated, which allowed us to look at tendencies over time. Fillion et al. (2006) [26] reported values of 113.9 ± 14.6 mmHg for mean SBP and 73.7 ± 11.0 mmHg for mean DBP in riverine communities in this region. The mean SBP and DBP values in our study were 126.4 ± 21.5 and 78.3 ± 14.1 mm Hg, respectively, in the 444 participants in the Tapajós River basin, which is higher than those found 10 years ago (*t*-test; *p* < 0.0001). In 2006, systolic hypertension (defined as SBP ≥ 140 mmHg) had a prevalence of 8% in these populations [26]. Using this definition of SBP, the prevalence would be 24% (CI: 21.1–27.3) in our study. Our data are in accordance with other data showing high prevalence of SAH in Amazonian riverine populations [43]. Thus, NCDs appear to be a chronic problem in this area.

A subgroup composed only of ‘presumably healthy’ subjects was created to avoid biasing our conclusions by including subjects with NCD diagnosis such as SAH and/or DM (Figure 3). This subgroup excluded participants with a diagnosis or history of chronic diseases such as CVDs, hypertension, or DM, as well as participants with known risk factors, such as those who smoked, drank alcohol, or had drug dependency or chronic treatment with drugs. This analysis allowed us to identify 298 participants who showed altered FBS and/or SAH but who had not been diagnosed or treated. This meant that more than half (58.1%) of all participants with high FBS and/or hypertension were not aware that these conditions could make them at-risk of NCDs. This suggested that there are many undiagnosed cases in Amazonian riverine populations (Figure 3).

It is essential to analyze genetic susceptibility to NCDs in order to effectively manage and treat these vulnerable populations. This study was the first to analyze the *APOE4* genotype as a biomarker of susceptibility to NCDs in riverine populations of the Amazon. The allelic frequency distribution in this population was 0.05, 0.77, and 0.18 for the ε2, ε3, and ε4 alleles, respectively. This is similar to the distribution described previously in other populations in South America (0.046, 0.767, and 0.187, respectively) [44]. Carrying the ε4 allele increases susceptibility to many NCDs, such as diabetes and hypertension [8,9,10], and to neurodegenerative disorders, such as Alzheimer’s disease [11], and it does not seem to be influenced by sex [4,45,46,47]. This analysis identified 246 individuals (32.2%; CI: 28.9–35.7) with this risk factor (Figure 4). Moreover, the presence of ApoE4 in addition to hypertension increases the risk of Alzheimer’s disease 1.5-fold [48]; in our work, 55.0% (CI: 48.5–61.3) of ε4 carriers had hypertension. Preventive strategies are especially important for these individuals in order to reduce the prevalence of NCDs in the Amazon.

The finding that Amazonian riverine population have an *APOE4* frequency similar to that of other populations suggests that environmental factors rather than increased genetic susceptibility is responsible for the high prevalence of NCDs.

Traditionally, the isolation of the Amazonian populations has been considered to be a protective factor against CVDs [49]. In addition, the traditional lifestyle in this region with a diet in which fish is the main source of protein (high in omega-3 fatty acids and low in saturated fats) is protective against CVDs [50]. Unfortunately, Amazonian riverine population currently faces two main problems that are acting as negative modulators: environmental contamination and the nutritional transition.

Both regions in the present work represent anthropogenic contamination of the Amazonian environment. The major small-scale gold-mining area in the Amazon is located in the Tapajós River basin and is responsible for chronic environmental mercury contamination (reviewed by [51]). Mercury is also found naturally in Amazonian soils and is concentrated in the environment by man-made large-scale projects such as dams. One of the largest dams in the world is located in Tucuruí (Figure 1), and human exposure to high levels of mercury was recently demonstrated in riverine population in this area [34]. Mercury is bioaccumulated and biomagnified through the food chain and by piscivorous fish, which are the species that are typically consumed the most by riverine population [52,53]. These fish show the highest levels of contamination [52,53]. A growing body of evidence suggests that mercury exposure can increase the risk of adverse cardiovascular impact in exposed populations [26,54,55,56,57,58]. Indeed, in Tapajós area blood pressure was significantly associated with the total mercury in hair, supporting mercury cardiovascular toxicity [26].

The nutritional transition is a more recent issue in the Amazon. In the last decade, governmental program has increased the purchasing power of individuals at a level that is sufficient to influence the nutritional status of these populations in both positive and negative ways [2]. One program is “Bolsa Familia” which provides an economic incentive for the poorest families to keep their children in school. There has been an increase in carbohydrate-rich, fatty, low-fiber foods in the diet of people in the Tapajós River basin, and an increase in the presence of radios, furniture, etc. in homes has led to more sedentary habits [5]. In other populations around the world, changes like these have been associated with increases in chronic diseases such as type 2 diabetes, hypertension, and CVDs [59,60,61,62,63].

Our limited knowledge about the current health status of Amazonian populations makes it difficult to develop efficacious healthcare strategies. The Brazilian National Public Healthcare System, known as the Unified Health System (Sistema Único de Saúde, SUS, in Portuguese), theoretically provides free access to primary, secondary, and tertiary healthcare to citizens [20]. However, the reality is very different for citizens who live in the Amazon. Prevention strategies that are tailored to the different profiles of Amazonian populations are needed to control NCDs and to hopefully reverse the trend towards an even higher prevalence of NCDs. Our results will better assist the development of these preventive strategies and governmental actions for adequate management of NCDs in the Amazon.

## 5. Conclusions

The Amazonian riverine population showed a high prevalence of altered FBS (impaired glucose tolerance or suspected diabetes mellitus) and/or SAH. The prevalence of these conditions were as high as their prevalence in the largest cities in the country (Rio de Janeiro and São Paulo) and were well above the prevalence found in Amazonian urban regions. More than half of the individuals with altered findings had never been diagnosed and were not being treated for their conditions. This could contribute to the finding of an increased mortality rate (up to 28% in the Northern region of Brazil) due to cardiovascular diseases.

About one-third of the participants had a genetic susceptibility (*APOE4*) to NCDs, which helps identify high-risk individuals. This prevalence is similar to that in other South American populations, suggesting that additional factors underlie the alterations detected in our study. We hypothesize that environmental contamination and nutritional transitions are two such factors.

Prevention strategies that are tailored to specific Amazonian populations are needed to control and even reverse the high prevalence of NCDs. Our results will help develop such preventive strategies and can help shape appropriate governmental actions to better identify and manage NCDs in the Amazon.

## Figures and Tables

**Table 1:** Demographic and clinical characteristics of the Amazonian riverine population that participated in this study. Data are presented as medians and interquartile intervals. Differences between sexes were analyzed by a binomial test for the number of participants and by the Mann–Whitney U test for the other parameters. The *p*-values are shown.

Characteristics	Total	Sex		Gender Difference *p*-Value
		*Women*	*Men*	
**Subjects, *n* (%)**	763 (100.0)	487 (63.8)	276 (36.2)	<0.0001 ^a^
**Age, years**	47 (34–57)	44 (31–55)	51 (40–62)	<0.0001 ^b^
**Height, cm**	155 (151–162)	152 (148–156)	164 (158–169)	<0.0001 ^b^
**Weight, kg**	64.3 (56.0–74.6)	61.7 (53.7–71.0)	69.3 (60.1–78.3)	<0.0001 ^b^
**BMI, kg/m^2^**	26.0 (23.3–29.6)	26.7 (23.3–30.4)	25.5 (23.3–28.5)	0.0032 ^b^
**FBS, mg/dL**	90 (81–103)	90 (81–106)	90 (80–100)	ns ^b^
**SBP, mmHg**	125 (113–139)	121 (110–137)	129 (120–142)	<0.0001 ^b^
**DBP, mmHg**	79 (70–88)	77 (69–86)	80 (73–89)	<0.0001 ^b^

Note: BMI, body mass index (calculated as weight in kilograms divided by the square of height in meters). FBS, fasting blood sugar; SBP, systolic blood pressure; DBP, diastolic blood pressure; ns, non-significant. ^a^ Binomial test; ^b^ Mann-Whitney *U* test.
